# The Hospital

**Published:** 1918-02-09

**Authors:** 


THE
### INSTITUTIONAL WORKER
**Being a Special Supplement to "The Hospital"**

### OUR BUREAU OF INFORMATION.

### Rules for Correspondents.

1. Every letter must be accompanied by the coupon to be cut from the back cover (inside page) of The Hospital, current issue, and must contain the name and address of the correspondent with pseudonym for publication if desired. Replies by post cannot be given save under exceptional circumstances at the Editor's discretion.

2. Letters from Approved Homes in reply to special needs published in the Bureau should state terms and full particulars, and be sent prepaid under cover to the Editor of the Bureau with name written across coupon for identification.

3. Proprietors of Homes which have not yet been entered on the List of Approved Homes, but have spare accommodation likely to suit special needs, are invited to write for an application form for registration. The fee for registration, which includes two announcements of the Home in the Bureau and other privileges, is 10s.

4. All communications to be addressed to the Editor of The Hospital, 28 Southampton Street, Strand, London, W.C. 2, and marked "Bureau of Information."

### A System of Timekeeping for the Nursing and Domestic Staff.

### Answer to December Question.

**By "Housekeeper."**

The question was:

**What is the most satisfactory method of checking the going out and coming in of the nursing and domestic staffs of an institution which has three outlets, none of which it is practicable to keep locked during the day, nor is it possible to keep porters on duty for the purpose?**

Most institutions have three outlets: accident, tradesmen's, and main entrance, but it is most unusual for the members of the staff to be allowed to use any entrance at will. If all the members of the staff are allowed to leave the building in their off-duty time, one of the best systems is for the head of each ward, either sister or charge nurse, to book each nurse off duty when she leaves the ward and book her on duty when she returns. Where the staff live in a building separated from the institution, everyone leaving the building should enter name and time immediately before going out and also the time of returning; a book large enough to be ruled off into spaces for this purpose saves a lot of trouble. A copy of all off-duty time allowed should be kept by matron, and the time-book checked daily or weekly. Any time entered in the book not corresponding with the regulation time should be inquired into at once.

Any member of the staff granted a theatre or extra pass of any description should be given a special pass dated for the day it is granted, with the time allowed written on it. On returning to the building the holder of a late pass should get it signed, either by the person who acts as door-keeper during the night or by the night sister. All special passes should be returned to matron's office after being used.

The domestic staff is much easier to control, as the numbers are fewer and the off-duty more regular, as a longer time is given once or twice a week instead of shorter leave daily. The domestic staff should report themselves to the housekeeper when going off duty, and again when returning, unless a book is kept for them to book themselves in and out the same as the nursing staff.

For the whole of the staff it should be insisted on as a point of honour that every care be taken to book time and date correctly.

### Extending a Hospital Engineer's Work.

### Answer to January Question.

**By "Caledonian."**

The question was:

**In what ways can a hospital engineer be useful in addition to his routine work of attending to the heating apparatus?**

In addition to the routine duties pertaining to the heating apparatus, the hospital engineer can render very useful service to his institution as a "handyman" in many ways. More especially is this necessary in a medium-sized hospital, where the work does not warrant the employment of an electrician and plumber, etc. By virtue of his training the engineer can use his knowledge in a variety of ways. If electric light is installed, he can take periodical readings of the meter, keeping a record showing comparative consumption for like periods in each year. In this connection it may be noted as a good plan to indicate foggy days and special reasons for a larger consumption than normal. Again, new electric lamps require fitting. This should only be done by the engineer, who can note the "life" of globes used. By this means a check can be kept on careless treatment.

Telephones for use from wards to office and other places inside the hospital can also be attended to, and small repairs to electric bells executed.

The *x*-ray installation, too, can very often benefit by attention from the engineer, who can advise as to the remedy when something goes wrong. Motor-ambulances, lifts, etc., may all be looked after by the engineer.

It will probably also be found advantageous to place the keeping of gas consumption and coal records in the care of the engineer, who should keep a comparative record as mentioned for electric light.

If there is no fireman regularly on duty the engineer can supervise the keeping of water hydrants in good condition, ready for any outbreak of fire. He may also give instruction to the nursing staff on the essential points to observe in "fire fighting."

Water will probably be obtained from a public supply by meter, and here, again, the engineer may be usefully employed in keeping a comparative record to check abnormal consumption and avoid waste—if obtained from a well the pumping apparatus will come under the engineer's care.

At many hospitals the engineer manufactures iron bed - cradles, splints, and the like, which are required urgently. This is a very real boon in these days of difficulty in the supply and transport of goods.

Where there is a pharmaceutical laboratory the vacuum-pans, tablet-machine, soda-water plant, grinding-mills, etc., all require some little attention at times, and a little adjustment by a mechanic, like the engineer, may save many pounds which outside help would cost.

Many other services, such as repairs to instruments, sterilisers, artificial limbs, etc., may be rendered by the engineer.

These suggestions are put forward not with the idea of imposing all the aforementioned duties on the engineer, but to give a rough indication to hospital authorities how they can utilise the spare time of this officer, so that his energies may be directed into useful channels, especially at the present time of labour shortage.

### FEBRUARY QUESTIONS.

### (1) Hot Dinner-Plates.

A hospital has four wards, each containing twenty beds. The dinners are served from one inadequate general kitchen and there is no arrangement for heating the plates. Each ward has a very tiny kitchen, which holds a small gas-ring. How can the plates be best heated?

### (2) How to Sterilise Rubber Gloves.

Describe the best method of sterilising rubber gloves, to be used during operations, to ensure their being perfectly sterilised with the minimum amount of damage to the rubber.

### RULES.

The following rules must be observed:—

1. Contributions must be written on one side of the paper. Brevity and terseness are desirable features. The MSS. must bear the name and address of the sender and be accompanied by coupon to be cut from the back cover (inside page) of the current issue of The Hospital. A pseudonym must be chosen if the name is not to be published.

2. Contributions must be addressed to the Editor of The Hospital, Institutional Worker Supplement, 28 & 29 Southampton Street, Strand, London, W.C. 2, should reach him before the end of the current month, and be marked in the left-hand corner "Facts and Figures."

**A minimum payment of Five Shillings will be made for each published answer.**

### QUESTIONS INVITED.

In connection with our Question Box, we offer every month five shillings each for the two best questions which are sent in for consideration. The questions may be on any institutional subject and concern any department, from the secretary's to the porter's, or the matron's to the domestic staff; the one essential is that they must be practical and deal with points that have a definite relation to hospital or institutional administration, upkeep, finance, or management. Points relating to artificers' work, housekeeping, the laundry, out-patients, and other practical matters will be welcome. It is hoped that thus, when difficult or doubtful points arise in the course of their work, institutional workers will be encouraged to put them in the form of a question and send them to the Editor, The Hospital Bureau of Information, 28 & 29 Southampton Street, Strand, W.C. 2, marked "Question Box."

The best questions will be published in due course and our readers will be given the opportunity of answering them. Thus all institutional workers may be able to co-operate with the view of helping the work and smoothing the difficulties of each other. In short, we want the Question Box of the *Institutional Worker* to be freely used by every worker habitually.

### ENQUIRIES AND ANSWERS.

### *THE SICK AND IN NEED.*

### Hospital for Spinal Curvature.

The child suffering from curvature of the spine would receive the necessary examination and treatment at the Royal National Orthopædic Hospital, 234 Great Portland Street, W.—R. S. F.

### *WAR MATTERS.*

### How to Become a Lecturer to a Red Cross Detachment.

We suggest that you write to the Secretary, V.A.D., British Red Cross Society, Devonshire House, Piccadilly, London, S.W., in regard to obtaining a lectureship to a Red Cross Detachment. —Parler.

### Helpers Wanted for Carpentry by the S.R.A,

The Surgical Requisites Association, 17 Mulberry Walk, Church Street, Chelsea, S.W. 3, are in urgent need of helpers for their carpenters' shops to enable them to meet the large demands made upon them, and we suggest that you communicate with the Hon. Secretary of the Association, at the above address.—S. M. R.

### How to Become a V.A.D.

Write to the Secretary, Voluntary Aid Detachment, Devonshire House, Piccadilly, London, S.W., who will furnish you with all the necessary information that you require.—K. M. C.

### *MISCELLANEOUS.*

### Electric-lamp Tops.

Yes, there is a market for worn-out electric lamps. We suggest that you apply to Messrs. Cardwell and Fenn, Ltd., electric-lamp specialists, of 36 Shaftesbury Avenue, London, W. If you will refer to our issue of July 10, 1915, you will find an instructive article upon this subject.—Economist.

### Treatment of Discharged Soldiers.

The question as to whether you should receive into your hospital discharged soldiers referred to you for treatment by various local Pensions Committees is one which your committee of management must decide. Certain hospitals are accepting these cases as ordinary civilian cases and declining the payment offered by the Ministry of Pensions, so that, pending a definite agreement, they may not be faced with the resulting difficulties, as, for instance, the question of paying the medical staff. The agreement recently arrived at between the British Hospitals Association and the Ministry of Pensions does not apparently represent the views of the hospitals generally, so that the difficulties are no nearer a solution. There is nothing, however, to prevent your management from deciding upon a course independently after consulting their medical staff.—Perplexed.

### *EMPLOYMENT AND TRAINING.*

### Institutional Housekeeping.

The Royal Victoria Infirmary, Queen Victoria Road, Newcastle-on-Tyne, accepts pupil-housekeepers for a period of six months, during which time a thorough knowledge of the work is obtained. Candidates must be certificated nurses and well recommended by their matrons. A salary at the rate of £15 a year is given, together with board, lodging, and washing. Lord Mayor Treloar Cripples' Hospital, Alton, Hants, and the South Devon and East Cornwall Hospital, Plymouth, both receive pupil-housekeepers. Application should be made to the matron in each case.—Chow.

### The Necessary Training of a Fully-trained Nurse.

Your experience as a war probationer will not count in any way towards the training necessary to obtain a certificate which would qualify you as a fully-trained nurse. The requirement is three years' consecutive training at a recognised training-school —*i.e.*, a hospital of 100 beds or over.—War-worker.

### *THE HOUSEKEEPERS' DEPARTMENT.*

### Potato Machine.

We would advise you to defer purchasing a potato-paring machine in view of the need for economy in all directions. The potato has a higher food value when cooked in the skin, and if before boiling the skin is cut around the long axis it is readily removed afterwards. Thus both labour and material can be saved and greater efficiency attained.—Norah.

### Meat Supply.

It is apparently true that the Ministry of Food has issued instructions to the butchers to supply their customers with only 50 per cent. of their purchases between certain dates last October, and pending compulsory rationing the Ministry desires the hospitals to reduce their requirements. It was not apparently intended that their supplies should be reduced by 50 per cent., for local Food Committees were instructed that the hospitals should have priority—that is, that their claim should be considered after the needs of the Admiralty and War Office have been satisfied. We suggest that you write to the Food Committee controlling the area in which your butcher is located.—Secretary.

**The Editor will be glad to receive correspondence and to consider contributions upon all subjects relating to institutional work which affect the welfare of institutional workers.**

